# *Bidens pilosa* Formulation Improves Blood Homeostasis and ***β***-Cell Function in Men: A Pilot Study

**DOI:** 10.1155/2015/832314

**Published:** 2015-03-18

**Authors:** Bun-Yueh Lai, Tzung-Yan Chen, Shou-Hsien Huang, Tien-Fen Kuo, Ting-Hsiang Chang, Chih-Kang Chiang, Meng-Ting Yang, Cicero Lee-Tian Chang

**Affiliations:** ^1^Chun-Yueh Biomedical Technology Co., Ltd., Taipei 116, Taiwan; ^2^Agricultural Biotechnology Research Center, Academia Sinica, 128 Academia Road, Section 2, Nankang, Taipei 115, Taiwan; ^3^Institute of Biotechnology, National Taiwan University, Taipei 106, Taiwan; ^4^Graduate Institute of Life Sciences, National Defense Medical Center, Taipei 114, Taiwan; ^5^Department of Cancer Biology and Medicine, China Medical University, Taichung 404, Taiwan; ^6^Department of Chinese Medicine, Buddhist Tzu Chi General Hospital, Hualien 970, Taiwan; ^7^Department of Veterinary Medicine, National Chung Hsing University, 250 Kuo-Kuang Road, Taichung 402, Taiwan

## Abstract

*B. pilosa* has long been purported to have antidiabetes activity, but despite the advancement in phytochemistry and animal models of diabetes, no human clinical trials have been conducted to date. Here, we evaluated the effect of a *B. pilosa* formulation on fasting blood glucose (FBG), fasting serum insulin, and glycosylated hemoglobin A1c (Hb_A1c_) in diabetic subjects. The *B. pilosa* formulation reduced the level of FBG and Hb_A1c_ in diabetics but increased fasting serum insulin in healthy subjects. Moreover, combination of *B. pilosa* formulation with antidiabetic drugs had better glycemic control in diabetics. The homeostatic model assessment (HOMA) data suggested that the antidiabetic activity of this formulation was via improvement of *β*-cell function. We also tested the safety of the *B. pilosa* formulation in healthy subjects and observed no obvious side effects. We conclude that *B. pilosa* has potential as an antidiabetes treatment.

## 1. Introduction 

Type 2 diabetes is a global health problem that carries a large economic burden. According to the International Diabetes Foundation 382 million people were diagnosed with diabetes in 2013 and this number is expected to rise to 592 million by 2035 [1]. Current oral antidiabetic drugs have unmet efficacy and undesirable side effects in patients often leading to lethal complications [2]. Therefore, continuing the search for new diabetes treatments is a priority.

Over 1200 plants are purported to have antidiabetic activity [3, 4]. Among them,* B. pilosa *has long been used as an antidiabetic herb in Asia, America, and Africa [5]. However, no clinical trial has ever evaluated the efficacy and safety of this herb [3, 6]. We and other groups have shown that* B. pilosa *has hypoglycemic activity in diabetic db/db mice and alloxan-treated mice [7–9]. Three polyynes from* B. pilosa *were found to have glucose-lowering activity [8, 9]. Among them, cytopiloyne identified from* B. pilosa *had better glucose-reducing activities in diabetic mice than the other two polyynes [9]. We also demonstrated that* B. pilosa *and cytopiloyne lowered blood glucose via insulin secretion and islet protection [4]. Further, mechanistic studies showed that cytopiloyne and, probably,* B. pilosa* exerted antidiabetic action via their regulation of *β*-cell function [4].

Despite some claims of human antidiabetic activity, there have been no modern clinical evaluations of* B. pilosa *in humans. In this study, we evaluated the efficacy and safety of a* B. pilosa *formulation in human diabetic and healthy subjects.

## 2. Materials and Methods 

### 2.1. Efficacy Pilot Study

Fourteen volunteers whose fasting blood glucose was more than 126 mg/dL and/or whose 2 h postmeal prandial blood glucose was more than 200 mg/dL were diagnosed as diabetics based on the American Diabetes Association criteria. They were grouped into 2 groups. One group, 6 diabetics, only consumed the* B. pilosa *formulation (probetacell) orally at a dose of 400 mg,* ter in die*, for 3 to 7 months. The other group, 8 diabetics, took antidiabetic drugs plus the* B. pilosa *formulation. Their blood samples were collected before and after their treatment. Biochemical parameters of the blood samples from both groups were determined (Table 1) based on the manufacturers' protocols. Briefly, triglyceride (TRIG), total cholesterol (TC), high density lipoprotein (HDL), low density lipoprotein (LDL), aspartate aminotransferase (AST), alanine aminotransferase (ALT), and blood urine nitrogen (BUN) were analyzed with 7600 Clinical Analyzer (Hitachi). Serum insulin was quantified with the ADVIA Centaur ELISA Kits (Siemens). Hb_A1c_ was measured using a DCA 2000 analyzer (Bayer). The* B. pilosa *formulation (probetacell) is a commercial functional food in Taiwan (Chun-Yueh Biomedical Technology Co., Ltd.) and HPLC was used to control the quality of the formulations (see Sup. Figure 2 in Supplementary Material available online at http://dx.doi.org/10.1155/2014/832314).

### 2.2. Safety Pilot Study

Blood from seven healthy volunteers was collected before and after they took the* B. pilosa *formulation (probetacell) orally at a daily dose of 400 mg per person,* ter in die*, for 3 months. The biochemical parameters (Table 2) of the blood samples were analyzed as above.

### 2.3. Statistical Analysis

Data from three independent experiments or more are presented as mean ± SEM. Student's *t*-test was used for statistical analysis of the differences between groups. A *P* value ( ^*^) of less than 0.05 was considered to be statistically significant.

## 3. Results and Discussion 

### 3.1. *B. pilosa* Formulation Improves Type 2 Diabetes via Promotion of *β*-Cell Function

Our group and others previously demonstrated that* B. pilosa *exerted antidiabetic activity in mouse models, so in this study we verified this effect in humans. First, we evaluated the beneficial effect of the* B. pilosa *formulation on subjects with type 2 diabetes. We found that those who only took the* B. pilosa *formulation had fasting blood glucose levels of 201.7 ± 83.3 and 123.3 ± 18.6, respectively, before and after treatment with the* B. pilosa *formulation (Table 1). Similarly, the diabetics had Hb_A1c_ levels of 9.1 ± 1.7 and 7.2 ± 0.7, respectively, before and after the treatment with the* B. pilosa *formulation (Table 1). The HOMA-IR and HOMA-*β* are commonly used to assess insulin resistance and *β*-cell function, respectively [10]. Treatment with the* B. pilosa *formulation significantly increased *β*-cell function of the participants as shown by the HOMA-*β* values. In contrast, the treatment did not affect their insulin resistance, as shown by the HOMA-IR values (Sup. Figure 1). Accordingly, the* B. pilosa *formulation boosted serum insulin level in healthy persons (Table 2). Besides, we tested the combination effect of the* B. pilosa *formulation. We found that those who only took antidiabetic drugs and the* B. pilosa *formulation had fasting blood glucose levels of 220 ± 70.9 and 150 ± 51.3, respectively, before and after the combination treatment (Table 1). However, the combination use of the* B. pilosa *formulation seemed better than its single use based on the data on the decreased ratio of fasting blood glucose and Hb_A1c_ (Table 1).

Overall, the data from this study are in good agreement with previous studies in mice [4] that suggested that* B. pilosa *enhanced insulin secretion and islet preservation via *β*-cell regulation.

### 3.2. *B. pilosa* Formulation Had No Obvious Side Effects

**Figure 1:**
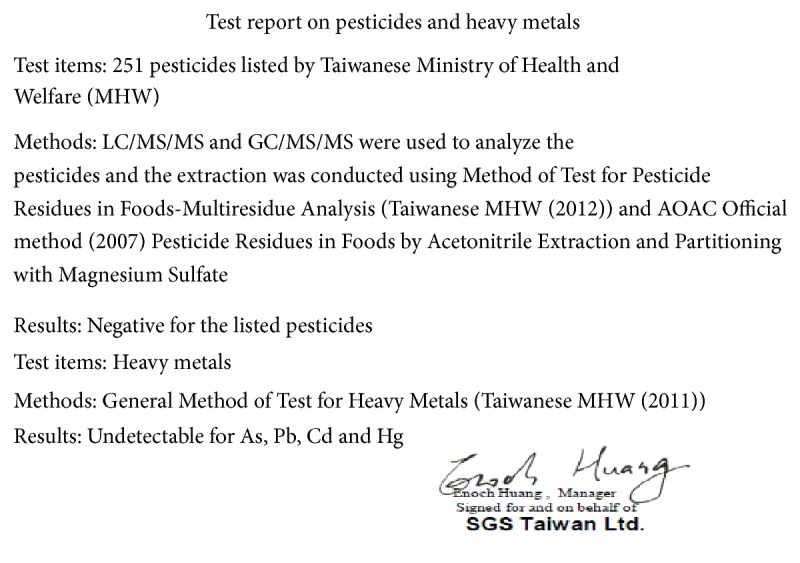
Report on the contamination of pesticides and heavy metals in the* B. pilosa *formulation used in this study. The content of the pesticides and heavy metals in the* B. pilosa *formulation was determined and certificated by SGS Taiwan Ltd.

Next, we assessed the 90-day safety of the* B. pilosa *formulation in 7 diabetes-free volunteers. We found that 90-day administration with the* B. pilosa *formulation showed no obvious adverse effects (Table 2). In addition, heavy metals (As, Pb, Cd, and Hg) and 251 pesticides in the* B. pilosa *formulation used in the study were determined and their concentrations are below the limit of detection (Figure 1 and Sup. Table 1). The Food and Agricultural Organization of the United Nations recognizes* B. pilosa *as a staple food [11]. The Ministry of Health and Welfare in Taiwan also allows its use as an ingredient in food for human consumption. Previous studies by our group and others found no toxicity of* B. pilosa *in mouse models [5, 6] and rats [12]. However, comprehensive scientific study of the safety of* B. pilosa *has not been conducted. In this work, clinical data suggest that* B. pilosa *at 400 mg,* ter in die*, has no noticeable toxicity (Table 2). Large-scale clinical trials on the efficacy and toxicology of* B. pilosa *in humans are required prior to its further medical use.

In summary, our clinical data demonstrated that the* B. pilosa *formulation had an antidiabetic action and no obvious side effects in humans. This action involves the regulation of *β*-cells.

## Supplementary Material

Fig. 1: Changes in HOMA-beta and HOMA-IR in diabetic subjects before and after treatment with B. pilosa formulation.
Fig. 2: HPLC profiles of three batches of Bidens pilosa formulation.
Table 1: 251 pesticides list by Taiwan Ministry of Health and Welfare.

## Figures and Tables

**Table 1:** Selected biochemical parameters of diabetic subjects after administration with *B. pilosa* formulation for 3 to 7 months.

Parameters^a^	Age (yr)	Diabetic history (yr)	Treatment time (m)	FBG (mg/dL)^b^				Hb_A1c_ (%)			
				Pretreatment	Posttreatment	Decreased ratio^c^	*P* value^d^	Pretreatment	Posttreatment	Decreased ratio^c^	*P* value^d^
BP without antidiabetic drugs^e^ (*n* = 6)	65.6 ± 10.5	7.0 ± 5.3	5.0 ± 2.0	201.7 ± 83.3	123.3 ± 18.6	0.33 ± 0.20	0.048	9.1 ± 1.7	7.2 ± 0.7	0.19 ± 0.07	0.033
BP with antidiabetic drugs^f^ (*n* = 8)	61.3 ± 11.6	12.4 ± 6.3	3.6 ± 0.9	220 ± 70.9	150 ± 51.3	0.31 ± 0.14	0.040	8.6 ± 0.6	7.7 ± 0.7	0.10 ± 0.05	0.012

^a^All data are presented as mean ± SD.

^b^FBG: fasting blood glucose.

^c^Decreased ratio = (value of pretreatment − value of posttreatment)/value of pretreatment.

^d^Data are presented as mean ± SD (standard deviation). Student's *t*-test was used for statistical analysis between pretreatment and posttreatment. The *P* values (<0.05) are considered statistically significant.

^e^Diabetic patients only consumed BP supplement. The number (*n*) of volunteers is indicated.

^f^Diabetic patients consumed antidiabetic drugs and BP supplement (combination therapy). These antidiabetic drugs included metformin (Glucophage) dominantly and acarbose (Glucobay), glibenclamide (Euglucon), glimepiride (Amaryl), and insulin (NovoMix 30 or NPH human insulin/Humulin).

**Table 2:** Selected biochemical parameters of healthy volunteers after administration with the *B. pilosa* formulation for 3 months.

Parameters^a^	Hb_A1c_ (%)	FBG (mg/dL)	PBG (mg/dL)	Fasting insulin (mU/L)	Postprandial insulin (mU/L)	TRIG (mg/dL)	TC (mg/dL)	HDL-c (mg/dL)	LDL-c (mg/dL)	AST (U/L)	ALT (U/L)	BUN (mg/dL)	Creatinine (mg/dL)
Pretreatment (*n* = 7)	5.4 ± 0.3	87.6 ± 2.3	111.6 ± 25.7	3.4 ± 1.4	12.5 ± 10.2	85.1 ± 36.0	168.4 ± 27.3	55.8 ± 10.6	86.4 ± 21.1	21.1 ± 7	15.7 ± 4.9	13 ± 3.1	0.8 ± 0.1
Posttreatment (*n* = 7)	5.4 ± 0.3	90 ± 6.2	115.1 ± 31.3	4.9 ± 7.7	23.5 ± 16.4	71.6 ± 24.5	161.1 ± 20.9	53.3 ± 7	86.4 ± 19.5	17 ± 2	13.6 ± 3.6	13.4 ± 2.8	0.8 ± 0.1
P value^b^	0.86	0.35	0.82	0.62	0.16	0.43	0.58	0.61	1	0.16	0.36	0.8	0.83

^a^Data from seven healthy volunteers are presented as mean ± SD (standard deviation). The number (*n*) of volunteers is indicated.

^b^Student's *t*-test is used to compare the parameters before and after the volunteers took the *B. pilosa* formulation at a daily dose of 400 mg per person, *ter in die*. No statistical significance is found.

## Abbreviations

Hb_A1c_: Glycosylated hemoglobin A1c
FBG: Fasting blood glucose
TRIG: Triglycerides
TC: Total cholesterol
HDL: High density lipoprotein
LDL: Low density lipoprotein
AST: Aspartate aminotransferase
ALT: Alanine aminotransferase
BUN: Blood urine nitrogen.
