# Supplementary material for: Bidens pilosa Formulation Improves Blood Homeostasis and β-Cell Function in Men: A Pilot Study
Source: Evid Based Complement Alternat Med. 2015 Mar 18;2015:832314. doi: 10.1155/2015/832314 (PMC4381681; doi:10.1155/2015/832314)
Supplement: Supplementary file 1 — Fig. 1: Changes in HOMA-beta and HOMA-IR in diabetic subjects before and after treatment with B. pilosa formulation. Fig. 2: HPLC profiles of three batches of Bidens pilosa formulation. Table 1: 251 pesticides list by Taiwan Ministry of Health and Welfare. [file 832314.f1.zip › Description.docx]

**Sup. Fig. 1.** Changes in HOMA-beta and HOMA-IR in diabetic subjects before and after treatment with *B. pilosa* formulation.

**Sup. Fig. 2 . HPLC profiles of three batches of *Bidens pilosa* formulation.**

Sup. Table 1. 251 pesticides list by Taiwan Ministry of Health and Welfare

HOMA-β and HOMA-IR

Blood samples of 8 diabetic subjects were collected and measured before and after

treatment with B. pilosa formulation. Their HOMA-β (A) and HOMA-IR (B) were

calculated from fasting blood glucose and serum insulin based on the following

formulae. The insulin resistance index (HOMA-IR) and β-cell function (HOMA-β)

assessed by the homeostasis model assessment (HOMA) were calculated as follows.

HOMA-IR = insulin (μU/mL) × glucose (mmol/L)/22.5 and HOMA-β = 20 × insulin

(μU/mL)/(glucose (mmol/L)-3.5).

HPLC

Three batches of the B. pilosa formulation were extracted, underwent RP-18 HPLC

column and detected with a UV detector at 230 nm.
