# Supplementary material for: Bidens pilosa Formulation Improves Blood Homeostasis and β-Cell Function in Men: A Pilot Study
Source: Evid Based Complement Alternat Med. 2015 Mar 18;2015:832314. doi: 10.1155/2015/832314 (PMC4381681; doi:10.1155/2015/832314)
Supplement: Supplementary file 1 — Fig. 1: Changes in HOMA-beta and HOMA-IR in diabetic subjects before and after treatment with B. pilosa formulation. Fig. 2: HPLC profiles of three batches of Bidens pilosa formulation. Table 1: 251 pesticides list by Taiwan Ministry of Health and Welfare. [file 832314.f1.zip › Sup Fig 2.doc]

Absorption (mAU)

Sup. Fig. 2.

0

200

400

600

800

0

200

400

600

800

50

0

10

20

30

40

0

200

400

600

800

**Batch 2**

**Batch 3**

**Batch 1**

Retention time (min)

1

3

2

1

3

2

1

3

2
