# Supplementary material for: Bidens pilosa Formulation Improves Blood Homeostasis and β-Cell Function in Men: A Pilot Study
Source: Evid Based Complement Alternat Med. 2015 Mar 18;2015:832314. doi: 10.1155/2015/832314 (PMC4381681; doi:10.1155/2015/832314)
Supplement: Supplementary file 1 — Fig. 1: Changes in HOMA-beta and HOMA-IR in diabetic subjects before and after treatment with B. pilosa formulation. Fig. 2: HPLC profiles of three batches of Bidens pilosa formulation. Table 1: 251 pesticides list by Taiwan Ministry of Health and Welfare. [file 832314.f1.zip › Sup Fig.1.doc]

**HOMA-β**

A

**0**

**20**

**40**

**60**

**P = 0.023**

**Pre-treatment**

**Post-treatment**

Sup. Fig. 1.

**Post-treatment**

**P = 0.98**

B

**HOMA-IR**

**Pre-treatment**

**0**

**1**

**2**

**3**

**4**

**5**
