# Supplementary material for: Bidens pilosa Formulation Improves Blood Homeostasis and β-Cell Function in Men: A Pilot Study
Source: Evid Based Complement Alternat Med. 2015 Mar 18;2015:832314. doi: 10.1155/2015/832314 (PMC4381681; doi:10.1155/2015/832314)
Supplement: Supplementary file 1 — Fig. 1: Changes in HOMA-beta and HOMA-IR in diabetic subjects before and after treatment with B. pilosa formulation. Fig. 2: HPLC profiles of three batches of Bidens pilosa formulation. Table 1: 251 pesticides list by Taiwan Ministry of Health and Welfare. [file 832314.f1.zip › Sup Table 1..doc]

Sup. Table 1. 251 pesticides list by Taiwan Ministruy of Health and Welfare

Test items and MDL list :	Unit : ppm (mg/kg)

| No. | Test item | CAS NO. | MDL |
| --- | --- | --- | --- |
| 1. | 2,4-D | 94‐75‐7 | 0.1 |
| 2. | 3-keto Carbofuran | 16709-30-1 | 0.05 |
| 3. | 3-OH carbofuran | 16655-82-6 | 0.05 |
| 4. | Abamectin | 71751‐41‐2 | 0.05 |
| 5. | Acephate | 30560-19-1 | 0.25 |
| 6. | Acetamiprid | 135410-20-7 | 0.05 |
| 7. | Alachlor | 15972‐60‐8 | 0.2 |
| 8. | Aldicarb | 116-06-3 | 0.15 |
| 9. | Aldicarb sulfone | 1646-88-4 | 0.05 |
| 10. | Aldicarb sulfoxide | 1646-87-3 | 0.15 |
| 11. | Aldrin | 309-00-2 | 0.05 |
| 12. | Allethrin | 584‐79‐2 | 0.25 |
| 13. | Alphacypermethrin | 67375-30-8 | 0.25 |
| 14. | Azinphos-methyl | 86-50-0 | 0.25 |
| 15. | Azoxystrobin | 131860-33-8 | 0.05 |
| 16. | Bendiocarb | 22781-23-3 | 0.05 |
| 17. | Benfluralin | 1861-40-1 | 0.05 |
| 18. | Benfuracarb | 82560‐54‐1 | 0.05 |
| 19. | Bensulfuron-methyl | 83055-99-6 | 0.05 |
| 20. | Bentazone | 25057‐89‐0 | 0.05 |
| 21. | Benthiazole | 21564-17-0 | 0.1 |
| 22. | α-BHC | 319-84-6 | 0.05 |
| 23. | β-BHC | 319-85-7 | 0.05 |
| 24. | δ-BHC | 319-86-8 | 0.05 |
| 25. | Bifenox | 42576-02-3 | 0.05 |
| 26. | Bifenthrin | 82657-04-3 | 0.15 |
| 27. | Bitertanol | 55179‐31‐2 | 0.1 |
| 28. | Boscalid | 188425‐85‐6 | 0.05 |
| 29. | Bromophos-ethyl | 4824-78-6 | 0.15 |
| 30. | Bromophos-methyl | 2104-96-3 | 0.15 |
| 31. | Bromopropylate | 18181-80-1 | 0.1 |
| 32. | Bupirimate | 41483-43-6 | 0.15 |
| 33. | Butachlor | 23184‐66‐9 | 0.05 |
| 34. | Butocarboxim | 34681-10-2 | 0.05 |
| 35. | Butralin | 33629-47-9 | 0.25 |
| 36. | Captafol | 2425-06-1 | 0.25 |
| 37. | Captan | 133-06-2 | 0.05 |
| 38. | Carbaryl | 63-25-2 | 0.05 |
| 39. | Carbendazim | 10605-21-7 | 0.05 |
| 40. | Carbofuran | 1563-66-2 | 0.05 |
| No. | Test item | CAS NO. | MDL |
| 41. | Carbophenothion | 786-19-6 | 0.1 |
| 42. | Carbosulfan | 55285‐14‐8 | 0.05 |
| 43. | Carpropamid | 104030-54-8 | 0.1 |
| 44. | Chinomethionat | 2439-01-2 | 0.05 |
| 45. | Chlorantraniliprole | 500008-45-7 | 0.1 |
| 46. | α-Chlordane | 5103-71-9 | 0.05 |
| 47. | β-Chlordane | 5103-74-2 | 0.05 |
| 48. | Chlorfenapyr | 122453-73-0 | 0.25 |
| 49. | Chlorfluazuron | 71422-67-8 | 0.15 |
| 50. | Chloropropylate | 5836-10-2 | 0.25 |
| 51. | Chlorothalonil | 1897-45-6 | 0.05 |
| 52. | Chlorpyrifos | 2921-88-2 | 0.05 |
| 53. | Chlorpyrifos-methyl | 5598-13-0 | 0.15 |
| 54. | Chlozolinate | 84332-86-5 | 0.05 |
| 55. | Clofentezine | 74115-24-5 | 0.1 |
| 56. | Clomazone | 81777-89-1 | 0.05 |
| 57. | Clothianidin | 210880‐92‐5 | 0.1 |
| 58. | Cyanofenphos | 13067-93-1 | 0.05 |
| 59. | Cyazofamid | 120116-88-3 | 0.05 |
| 60. | Cyclosulfamuron | 136849-15-5 | 0.05 |
| 61. | Cyflumetofen | 400882-07-7 | 0.1 |
| 62. | Cyfluthrin | 68359-37-5 | 0.05 |
| 63. | Cyhalothrin | 91465-08-6 | 0.05 |
| 64. | Cymoxanil | 57966-95-7 | 0.1 |
| 65. | Cypermethrin | 52315-07-8 | 0.25 |
| 66. | Cyproconazole | 94361‐06‐5 | 0.05 |
| 67. | Cyprodinil | 121552-61-2 | 0.1 |
| 68. | o,p'-DDT | 789-02-6 | 0.05 |
| 69. | p,p'-DDD | 72-54-8 | 0.15 |
| 70. | p,p'-DDE | 72-55-9 | 0.05 |
| 71. | p,p'-DDT | 50-29-3 | 0.05 |
| 72. | Deltamethrin | 52918-63-5 | 0.05 |
| 73. | Demeton-S-methyl | 919-86-8 | 0.15 |
| 74. | Diazinon | 333-41-5 | 0.1 |
| 75. | Dichlofluanid | 1085-98-9 | 0.05 |
| 76. | Dichlorvos | 62-73-7 | 0.05 |
| 77. | Dicloran | 99-30-9 | 0.05 |
| 78. | Dicofol | 115-32-2 | 0.25 |
| 79. | Dicrotophos | 141‐66‐2 | 0.05 |
| 80. | Dieldrin | 60-57-1 | 0.05 |
| Test items and MDL list (cont.) : Unit : ppm (mg/kg) | | | |
| No. | Test item | CAS NO. | MDL |
| 81. | Difenoconazole | 119446-68-3 | 0.25 |
| 82. | Diflubenzuron | 35367-38-5 | 0.05 |
| 83. | Dimethenamid | 87674-68-8 | 0.1 |
| 84. | Dimethoate | 60-51-5 | 0.05 |
| 85. | Dimethomorph | 110488‐70‐5 | 0.05 |
| 86. | Diniconazole | 83657-24-3 | 0.15 |
| 87. | Dinitramine | 29091-05-2 | 0.05 |
| 88. | Diphenamid | 957-51-7 | 0.1 |
| 89. | Disulfoton | 298-04-4 | 0.05 |
| 90. | Ditalimfos | 5131-24-8 | 0.15 |
| 91. | Edifenphos | 17109‐49‐8 | 0.05 |
| 92. | α-Endosulfan | 959-98-8 | 0.05 |
| 93. | β-Endosulfan | 33213-65-9 | 0.05 |
| 94. | Endosulfan sulfate | 1031-07-8 | 0.05 |
| 95. | Endrin | 72-20-8 | 0.05 |
| 96. | EPN | 2104-64-5 | 0.05 |
| 97. | Epoxiconazole | 133855-98-8 | 0.35 |
| 98. | Esfenvalerate | 66230-04-4 | 0.1 |
| 99. | Ethion | 563-12-2 | 0.05 |
| 100. | Ethirimol | 23947-60-6 | 0.1 |
| 101. | Ethoprophos | 13194-48-4 | 0.05 |
| 102. | Etoxazole | 153233-91-1 | 0.1 |
| 103. | Etrimfos | 38260‐54‐7 | 0.25 |
| 104. | Famoxadone | 131807-57-3 | 0.1 |
| 105. | Fenamiphos | 22224-92-6 | 0.05 |
| 106. | Fenarimol | 60168-88-9 | 0.1 |
| 107. | Fenazaquin | 120928-09-8 | 0.05 |
| 108. | Fenbuconazole | 114369‐43‐6 | 0.5 |
| 109. | Fenitrothion | 122-14-5 | 0.05 |
| 110. | Fenobucarb | 3766-81-2 | 0.05 |
| 111. | Fenoxycarb | 79127-80-3 | 0.1 |
| 112. | Fenpropathrin | 39515-41-8 | 0.4 |
| 113. | Fenpropimorph | 67564-91-4 | 0.5 |
| 114. | Fenpyroximate | 134098-61-6 | 0.05 |
| 115. | Fensulfothion | 115-90-2 | 0.25 |
| 116. | Fenthion | 55-38-9 | 0.1 |
| 117. | Fenvalerate | 51630-58-1 | 0.15 |
| 118. | Fipronil | 120068-37-3 | 0.005 |
| 119. | Flazasulfuron | 104040‐78‐0 | 0.05 |
| 120. | Flonicamid | 158062-67-0 | 0.1 |
|  | | | |
| No. | Test item | CAS NO. | MDL |
| 121. | Fluazifop-P-butyl | 69806-50-4 | 0.05 |
| 122. | Flucythrinate | 70124-77-5 | 0.25 |
| 123. | Fludioxonil | 131341-86-1 | 0.1 |
| 124. | Flufenoxuron | 101463-69-8 | 0.05 |
| 125. | Fluopicolide | 239110-15-7 | 0.1 |
| 126. | Flusilazole | 85509-19-9 | 0.05 |
| 127. | Flutolanil | 66332-96-5 | 0.05 |
| 128. | Flutriafol | 76674-21-0 | 0.05 |
| 129. | Fluvalinate | 102851-06-9 | 0.25 |
| 130. | Fonofos | 944-22-9 | 0.1 |
| 131. | Formothion | 2540-82-1 | 0.15 |
| 132. | Halfenprox | 111872-58-3 | 0.25 |
| 133. | Haloxyfop-methyl | 69806-40-2 | 0.05 |
| 134. | Heptachlor | 76-44-8 | 0.25 |
| 135. | Heptachlor epoxide | 1024-57-3 | 0.05 |
| 136. | Heptenophos | 23560-59-0 | 0.05 |
| 137. | Hexaconazole | 79983-71-4 | 0.1 |
| 138. | Hexaflumuron | 86479-06-3 | 0.25 |
| 139. | Hexazinone | 51235-04-2 | 0.35 |
| 140. | Hexythiazox | 78587-05-0 | 0.25 |
| 141. | Imibenconazole | 86598‐92‐7 | 0.25 |
| 142. | Imidacloprid | 138261-41-3 | 0.25 |
| 143. | Indoxacarb | 144171-61-9 | 0.05 |
| 144. | Iprobenfos | 26087-47-8 | 0.15 |
| 145. | Iprodione | 36734-19-7 | 0.25 |
| 146. | Isazofos | 42509-80-8 | 0.05 |
| 147. | Isofenphos | 25311‐71‐1 | 0.1 |
| 148. | Isoprocarb | 2631-40-5 | 0.05 |
| 149. | Isoprothiolane | 50512-35-1 | 0.1 |
| 150. | Isoxathion | 18854-01-8 | 0.25 |
| 151. | Kresoxim-methyl | 143390‐89‐0 | 0.05 |
| 152. | Lindane | 58-89-9 | 0.05 |
| 153. | Lufenuron | 103055‐07‐8 | 0.05 |
| 154. | Malathion | 121-75-5 | 0.05 |
| 155. | Mefenacet | 73250‐68‐7 | 0.05 |
| 156. | Mephosfolan | 950-10-7 | 0.1 |
| 157. | Mepronil | 55814‐41‐0 | 0.05 |
| 158. | Metaflumizon | 139968-49-3 | 0.1 |
| 159. | Metconazole | 125116‐23‐6 | 0.05 |
| 160. | Methacrifos | 62610-77-9 | 0.1 |
| Test items and MDL list (cont.) : Unit : ppm (mg/kg) | | | |
| No. | Test item | CAS NO. | MDL |
| 161. | Methamidophos | 10265-92-6 | 0.1 |
| 162. | Methidathion | 950-37-8 | 0.1 |
| 163. | Methiocarb | 2032-65-7 | 0.05 |
| 164. | Methomyl | 16752-77-5 | 0.05 |
| 165. | Methoxyfenozide | 161050-58-4 | 0.1 |
| 166. | Methyl pentachlorophenyl sulfide | 1825-19-0 | 0.05 |
| 167. | Metolachlor | 51218-45-2 | 0.05 |
| 168. | Metolcarb | 1129-41-5 | 0.05 |
| 169. | Metribuzin | 21087‐64‐9 | 0.25 |
| 170. | Mevinphos | 7786-34-7 | 0.1 |
| 171. | Mirex | 2385-85-5 | 0.05 |
| 172. | Molinate | 2212-67-1 | 0.05 |
| 173. | Monocrotophos | 6923-22-4 | 0.1 |
| 174. | Myclobutanil | 88671-89-0 | 0.25 |
| 175. | Napropamide | 15299-99-7 | 0.05 |
| 176. | Nuarimol | 63284-71-9 | 0.25 |
| 177. | Omethoate | 1113-02-6 | 0.25 |
| 178. | Oxadiazon | 19666‐30‐9 | 0.05 |
| 179. | Oxadixyl | 77732-09-3 | 0.35 |
| 180. | Oxamyl | 23135-22-0 | 0.05 |
| 181. | Oxycarboxin | 5259-88-1 | 0.05 |
| 182. | Oxyfluorfen | 42874-03-3 | 0.15 |
| 183. | Paclobutrazol | 76738-62-0 | 0.05 |
| 184. | Parathion | 56-38-2 | 0.05 |
| 185. | Parathion-methyl | 298-00-0 | 0.05 |
| 186. | Penconazole | 66246-88-6 | 0.1 |
| 187. | Pencycuron | 66063‐05‐6 | 0.05 |
| 188. | Pendimethalin | 40487‐42‐1 | 0.05 |
| 189. | Pentachloroaniline | 527-20-8 | 0.05 |
| 190. | Permethrin | 52645-53-1 | 0.25 |
| 191. | Phenthoate | 2597-03-7 | 0.1 |
| 192. | Phorate | 298-02-2 | 0.05 |
| 193. | Phosalone | 2310-17-0 | 0.15 |
| 194. | Phosmet | 732-11-6 | 0.1 |
| 195. | Phosphamidon | 13171-21-6 | 0.25 |
| 196. | Phthalide | 27355-22-2 | 1 |
| 197. | Pirimicarb | 23103‐98‐2 | 0.05 |
| 198. | Pirimiphos-ethyl | 23505-41-1 | 0.05 |
| 199. | Pirimiphos-methyl | 29232-93-7 | 0.05 |
| 200. | Pretilachlor | 51218-49-6 | 0.25 |
|  | | | |
| No. | Test item | CAS NO. | MDL |
| 201. | Prochloraz | 67747-09-5 | 0.15 |
| 202. | Procymidone | 32809-16-8 | 0.25 |
| 203. | Profenophos | 41198-08-7 | 0.1 |
| 204. | Promecarb | 2631-37-0 | 0.05 |
| 205. | Propanil | 709‐98‐8 | 0.05 |
| 206. | Propaphos | 7292‐16‐2 | 0.05 |
| 207. | Propargite | 2312-35-8 | 0.05 |
| 208. | Propiconazole | 60207-90-1 | 0.15 |
| 209. | Propoxur | 114-26-1 | 0.05 |
| 210. | Prothiofos | 34643-46-4 | 0.05 |
| 211. | Pyraclofos | 77458-01-6 | 0.15 |
| 212. | Pyraclostrobin | 175013-18-0 | 0.05 |
| 213. | Pyrazophos | 13457-18-6 | 0.25 |
| 214. | Pyridaben | 96489-71-3 | 0.25 |
| 215. | Pyridaphenthion | 119-12-0 | 0.5 |
| 216. | Pyrifenox | 88283-41-4 | 0.15 |
| 217. | Pyriproxyfen | 95737‐68‐1 | 0.05 |
| 218. | Pyroquilon | 57369‐32‐1 | 0.05 |
| 219. | Quinalphos | 13593-03-8 | 0.1 |
| 220. | Quinoxyfen | 124495-18-7 | 0.25 |
| 221. | Quintozene(PCNB) | 82-68-8 | 0.1 |
| 222. | Quizalofop-ethyl | 76578-14-8 | 0.05 |
| 223. | Salithion | 3811-49-2 | 0.1 |
| 224. | Spirodiclofen | 148477-71-8 | 0.1 |
| 225. | Tebuconazole | 107534‐96‐3 | 0.05 |
| 226. | Tebufenozide | 112410-23-8 | 0.1 |
| 227. | Tebufenpyrad | 119168‐77‐3 | 0.1 |
| 228. | Teflubenzuron | 83121‐18‐0 | 0.05 |
| 229. | Tepraloxydim | 149979-41-9 | 0.1 |
| 230. | Terbufos | 13071-79-9 | 0.05 |
| 231. | Tetraconazole | 112281‐77‐3 | 0.05 |
| 232. | Tetradifon | 116-29-0 | 0.1 |
| 233. | Tetramethrin | 7696‐12‐0 | 0.05 |
| 234. | Thiabendazole | 148-79-8 | 0.05 |
| 235. | Thiacloprid | 111988-49-9 | 0.1 |
| 236. | Thiamethoxam | 153719-23-4 | 0.05 |
| 237. | Thiobencarb | 28249‐77‐6 | 0.05 |
| 238. | Thiodicarb | 59669-26-0 | 0.05 |
| 239. | Tolfenpyrad | 129558‐76‐5 | 0.05 |
| 240. | Triadimefon | 43121-43-3 | 0.05 |
| Test items and MDL list (cont.) : Unit : ppm (mg/kg) | | | |
| No. | Test item | CAS NO. | MDL |
| 241. | Triadimenol | 55219-65-3 | 0.25 |
| 242. | Triazophos | 24017-47-8 | 0.1 |
| 243. | Trichlorfon | 52-68-6 | 0.25 |
| 244. | Tricyclazole | 41814-78-2 | 0.1 |
| 245. | Trifloxystrobin | 141517‐21‐7 | 0.05 |
| 246. | Triflumizole | 99387-89-0 | 0.1 |
|  | | | |
| No. | Test item | CAS NO. | MDL |
| 247. | Trifluralin | 1582-09-8 | 0.05 |
| 248. | Triforine | 26644-46-2 | 0.1 |
| 249. | Vamidothion | 2275-23-2 | 0.25 |
| 250. | Vinclozolin | 50471-44-8 | 0.05 |
| 251. | XMC | 2655-14-3 | 0.05 |
